# Supplementary material for: Structure of a MacAB-like efflux pump from Streptococcus pneumoniae
Source: Nat Commun. 2018 Jan 15;9:196. doi: 10.1038/s41467-017-02741-4 (PMC5768738; doi:10.1038/s41467-017-02741-4)
Supplement: Supplementary file 1 — Supplementary Information [file 41467_2017_2741_MOESM1_ESM.pdf]

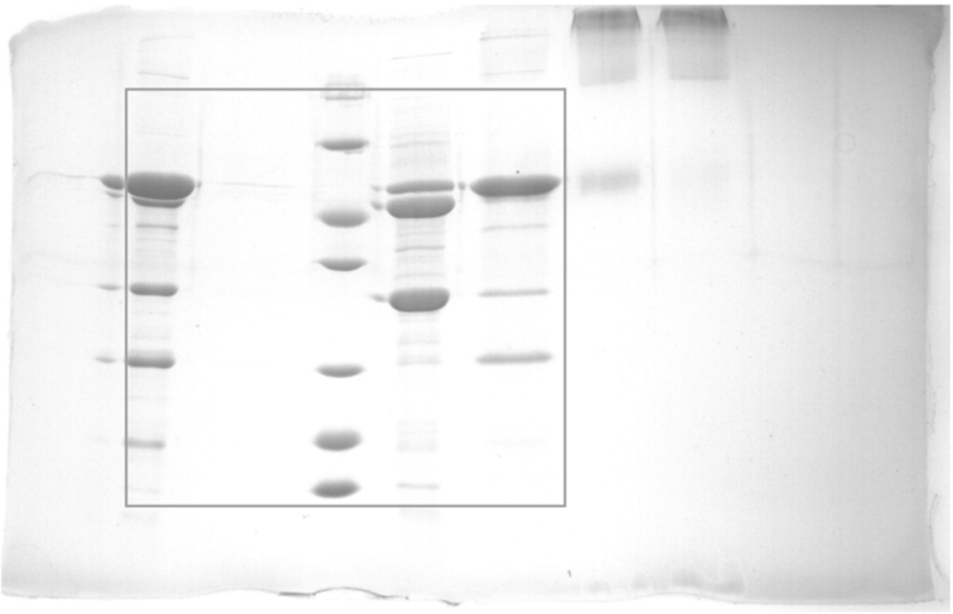

Supplementary Figure 1 The full SDS-PAGE image of Figure 3a. The area within the rectangle was adopted for Figure 3a.

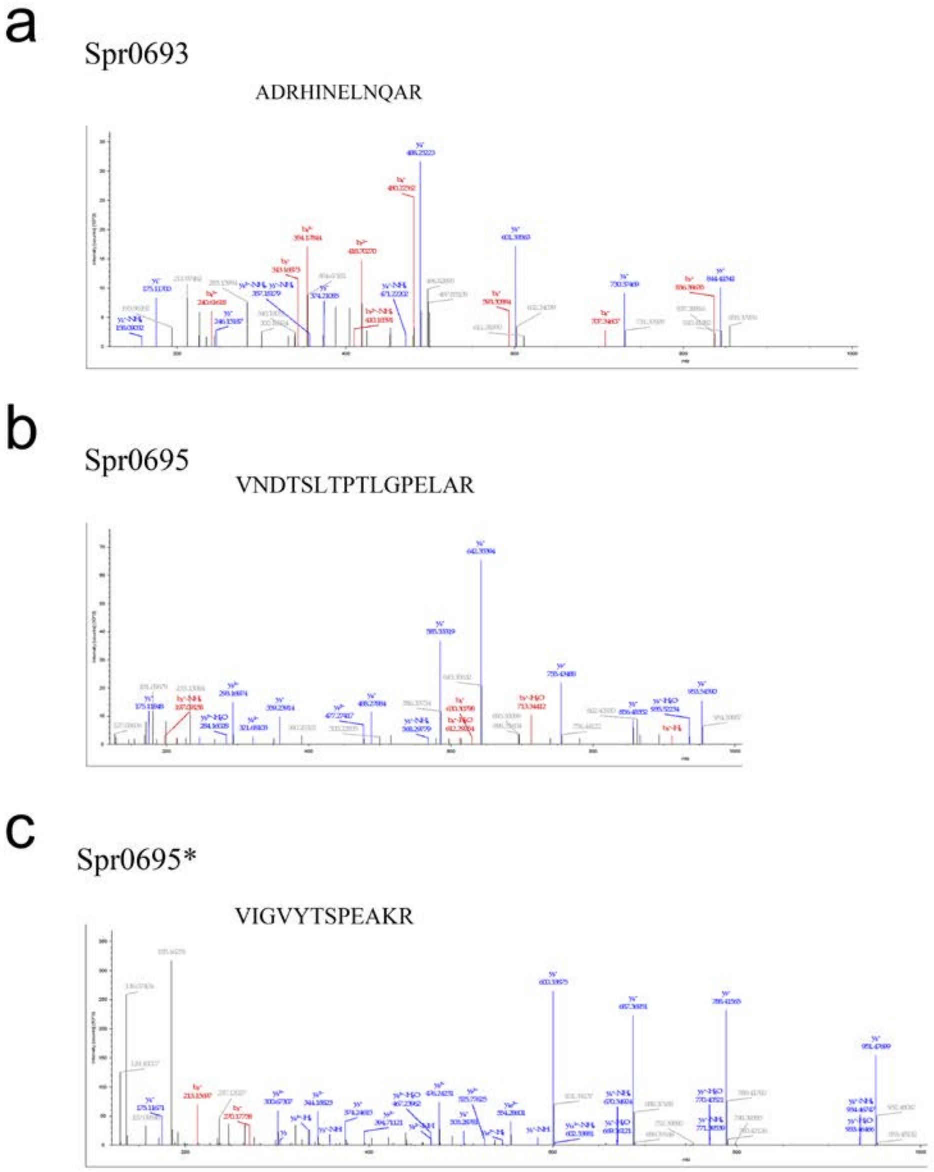

Supplementary Figure 2 Mass spectrometry identification of proteins in the pull-down assays. The bands on the SDS-PAGE gel were retrieved, lysed by trypsin and then analyzed by Mass spectrometry. The representative mass profile of one peptide from each protein is shown, with the corresponding sequence labeled on the top.

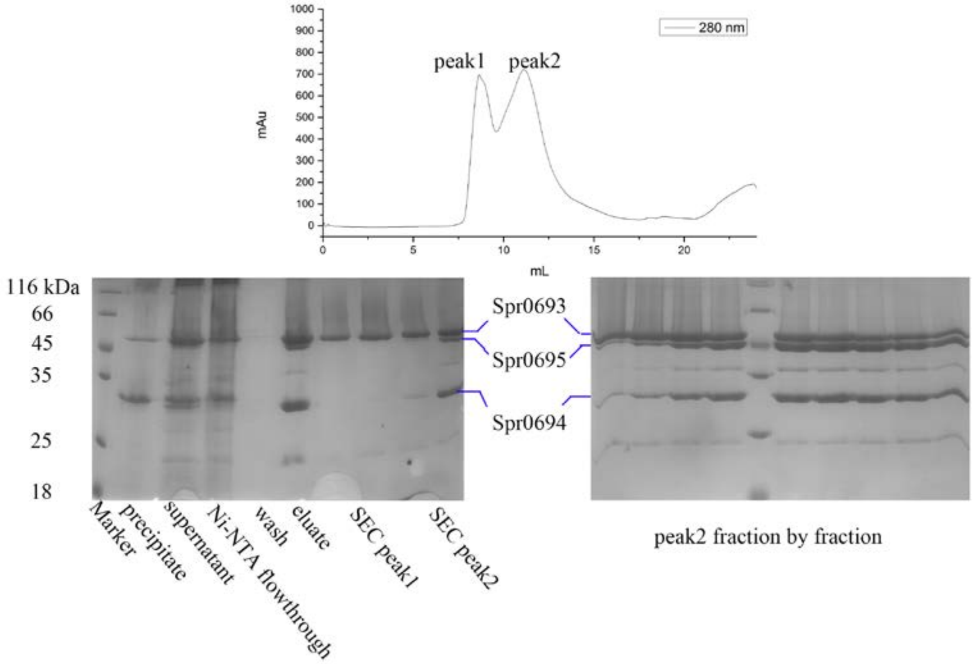

Supplementary Figure 3 Size-exclusion chromatography profile with co-purification of Spr0693-0694-0695.

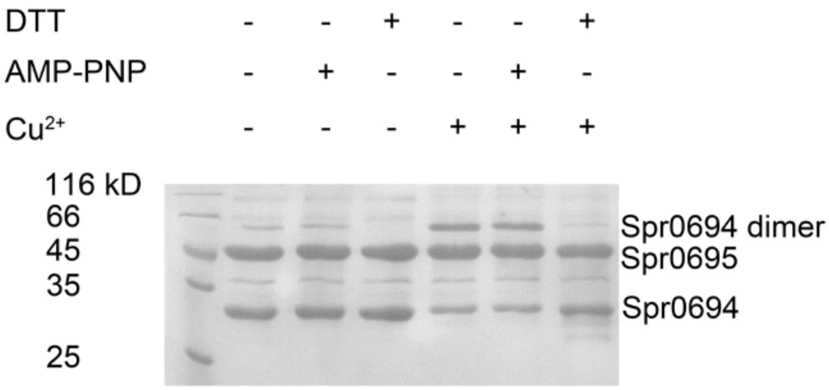

Supplementary Figure 4 The disulfide-crosslinking experiments of Spr0694$_{G173C}$-0695 mutant at different conditions. The reaction mix was separated by SDS-PAGE. Molecular masses are indicated on the left and the corresponding proteins are indicated on the right.

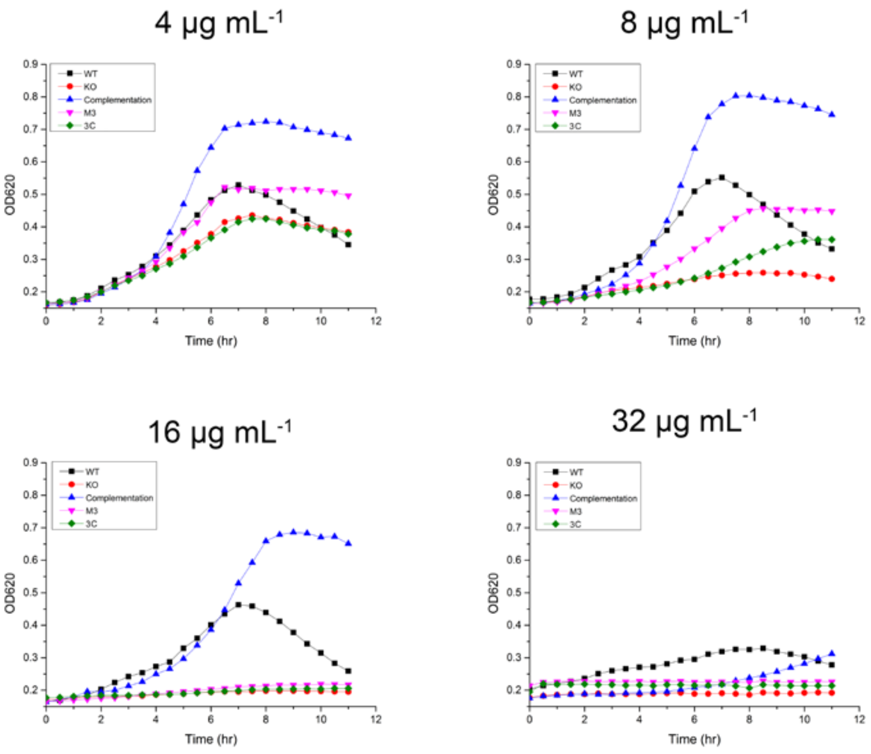

Supplementary Figure 5 Growth curves of *S. pneumoniae* R6 and mutants in the presence of LL-37 at different concentrations.

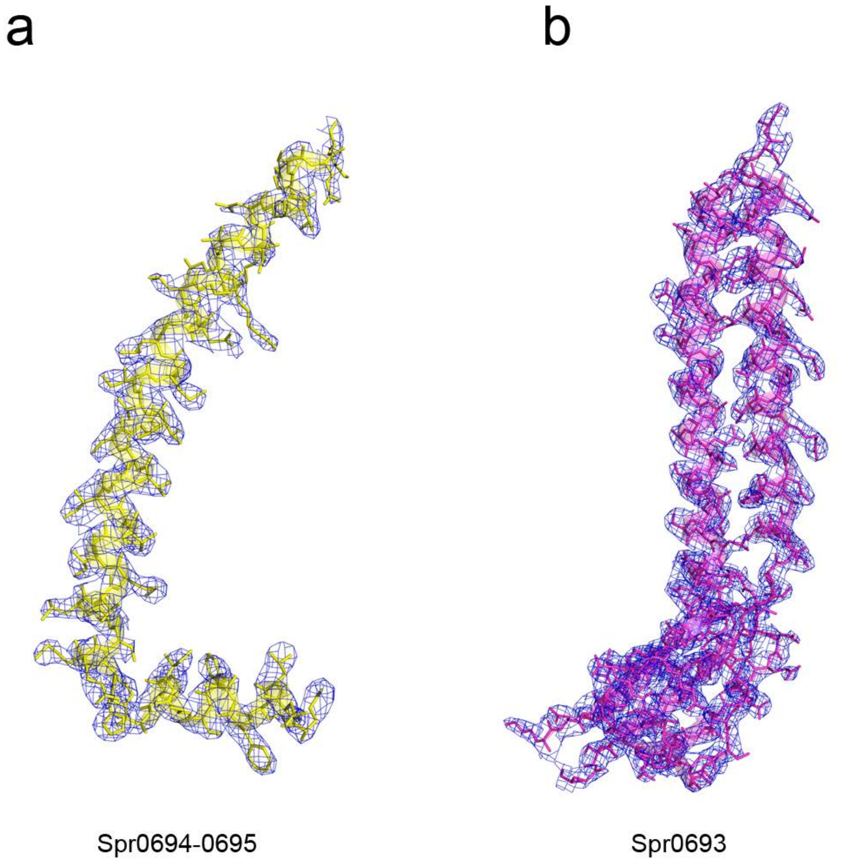

Supplementary Figure 6 A portion of the *2Fo-Fc* electron density map of Spr0694-0695 and Spr0693, countered at 1.5 σ. **(a)** Electron density map of TM1 of Spr0695. **(b)** Electron density map of the α-helical hairpin and lipoyl domains of Spr0693 monomer.

Supplementary Table 1 Bacterial strains, plasmids and primers used in this study

Strains, plasmids or primers	Description	Source
**Strains**		
WT	*S. pneumoniae* R6; *spr0693-0694-0695*$^{+}$; Kan^{s}	[1]
KO	*S. pneumoniae* R6 derivative; Δ*spr0693-0694-0695*::Janus cassette; Kan^{r}	This study
Complementation	KO strain transformed with pJWV25-*spr0693-0694-0695*; Kan^{r}, Tet^{r}	This study
M3	KO strain transformed with pJWV25-M3; Kan^{r}, Tet^{r}	This study
3C	KO strain transformed with pJWV25-3C; Kan^{r}, Tet^{r}	This study
**Plasmids**		
pJWV25	amp^{r}, tet^{r}, bgaA, P_{czcD}; integrate Zn^{2+}-controlled target genes by double cross-over at the *S. pneumoniae* bgaA locus.	[2]
pJWV25-*spr0693-0695*	amp^{r}, tet^{r}, bgaA, P_{czcD}-*spr0693-0694-0695*	This study
pJWV25-M3	amp^{r}, tet^{r}, bgaA, P_{czcD}-*spr0693-0694-0695*$_{K207A\text{-}R208A\text{-}K210A}$	This study
pJWV25-3C	amp^{r}, tet^{r}, bgaA, P_{czcD}-*spr0693-0694-0695* $_{D49C\text{-}R208C/K52C\text{-}E205C/E374C\text{-}K207C}$	This study
**Primers for MICs**	**Nucleotide sequence (5' to 3')**	
Up-F	GCGATATAACCAGCGCCTAGAATGGCAACTG	This study
Up-R	CAAACTCTGTGCTCGAGCACTCGCAGCACCGATTGCTGCATAC	This study
Down-F	CAGCATTATCCTCTAGAGAAGTTGGAGTATCAATCCCAGTTGCCC	This study
Down-R	GCGTAGCCAAGAGCTGTCGCATAGTTAAGAAG	This study
JC-F	GGTGCTGCGAGTGCTCGAGCACAGAGTTTGTAGAAACGCAAAAAGG	This study
JC-R	GATTGATACTCCAACTTCTCTAGAGGATAATGCTGAAAACTCCTTGAAG	This study
pJWV25-F	TGATAAGCTGAGCGCCGGTCGCTACCA	This study
pJWV25-R	CTTTTTCTTCATCTTGTCATCGTCATCCTTGTAGTCG	This study
pJWV25-Spr0693-F	GACCGGCGCTCAGCTTATCATTCATAACGAAGGGCTTC	This study
pJWV25-Spr0695-R	GACGATGACAAGATGAAGAAAAAGAATGGTAAAG	This study
Spr0695$_{K207A\text{-}R208A\text{-}K210A}$-F	GGAGGCTGCAGCGTCAGCAATATATGGGTTTGGTG	This study
Spr0695$_{K207A\text{-}R208A\text{-}K210A}$-R	CCCATATATTGCTGACGCTGCAGCCTCCGGACTAGTA	This study
Spr0695$_{D49C\text{-}K52C}$-F	CAAGTCAATAAATGTATGACTTGCTCTCAGAAAAATATTAGCGTC	This study
Spr0695$_{D49C\text{-}K52C}$-R	GCTAATATTTTTCTGAGAGCAAGTCATACATTTATTGACTTGACGAGATAGG	This study
Spr0695$_{E205C\text{-}K207C\text{-}R208C}$-F	GTTTATACTAGTCCGTGTGCTTGTTGCTCAAAAATATATGGGTTTGG	This study

Spr0695$_{E205C-K207C-R208C}$-R	CCATATATTTTTGAGCAACAAGCACACGGACTAGTATAAACCCCAATG	This study
Spr0695$_{E374C}$-F	GCAAGGTTTAATATGCGGTATAGAAGTTGGAGTATCAATC	This study
Spr0695$_{E374C}$-R	CCAACTTCTATACCGCATATTAAACCTTGCAGTAACAAAC	This study
**Primers for protein preparation**	**Nucleotide sequence (5’ to 3’)**	
pBAD-Spr0694-0695-F1	CATCATGGTACCATGAAGCAACTAATTAGTCTAAAAAATATCTTCAG	This study
pBAD-Spr0694-0695-R1	CTAATTAGTTGCTTCATGGTACCATGATGATGATGATGATGAGAACC	This study
pBAD-Spr0694-0695-F2	CGTTATGAATGATGAAGCTTGGGCCCGAACAAAAACTC	This study
pBAD-Spr0694-0695-R2	GGCCCAAGCTTCATCATTCATAACGAAGGGCTTCAATTGGATC	This study
Spr0694$_{E170Q}$-F	GCGGATCAGCCGACAGGAGCCTTGGATACC	This study
Spr0694$_{E170Q}$-R	CTGTCGGCTGATCCGCTAGGATAATAGAAGG	This study
Spr0694$_{G173C}$-F	CCGACATGTGCCTTGGATACCAAAACAGGTAAC	This study
Spr0694$_{G173C}$-R	CCAAGGCACATGTCGGTTCATCCGCTAGG	This study
Spr0695$_{K207A-R208A-K210A}$-F	GGAGGCTGCAGCGTCAGCAATATATGGGTTTGGTG	This study
Spr0695$_{K207A-R208A-K210A}$-R	CCCATATATTGCTGACGCTGCAGCCTCCGGACTAGTA	This study
pET29b-Spr0693-0694-0695-F1	GAAGGAGATATACATATGAAGAAAAAGAATGGTAAAGCTAAAAAGTG	This study
pET29b-Spr0693-0694-0695-R1	CCATTCTTTTTCTTCATATGTATATCTCCTTCTTAAAGTTAAACAAAATTATTTCTAG	This study
pET29b-Spr0693-0694-0695-F2	CCTTCGTTATGAACACCACCACCACCACCACTGAGATC	This study
pET29b-Spr0693-0694-0695-R2	TGGTGGTGGTGTTCATAACGAAGGGCTTCAATTGGATC	This study
pET29b-Spr0693-His-F	TGAAGCAACTAATCACCACCACCACCACCACTGAGATCC	This study
pET29b-Spr0693-His-R	CTCAGTGGTGGTGGTGGTGGTGATTAGTTGCTTCATCAGTCTTCACCTCTTTTCC	This study
pET29b-Spr0693-StrepII-F	CAACTAATTGGTCTCACCCCCAATTTGAGAAGTGAGATCCGGCTGCTAACAAAGCC	This study
pET29b-Spr0693-StrepII-R	GGATCTCACTTCTCAAATTGGGGGTGAGACCAATTAGTTGCTTCATCAGTCTTCACCTC	This study
Spr0693$_{34-399}$-F	GGAGATATACATATGAGACAACCTTCTCAGACTGCTCTAA	This study
Spr0693$_{34-399}$-R	GTGGTGGTGGTGGTGGTGATTAGTTGCTTCATCAGTCTTC	This study
Spr0693$_{59-324}$-F	GAAGGAGATATACATATGTCTGTTTTATTGTCAGGGACAGTA	This study
Spr0693$_{59-324}$-R	CAGTGGTGGTGGTGGTGGTGCTTAGTTTTGCTTTTAACCT	This study

	C	
Spr0693$_{70-237}$-F	GAAGGAGATATACATATGAATGAACAATATGTTTATTTTG	This study
Spr0693$_{70-237}$-R	CAGTGGTGGTGGTGGTGGTGCTTGACTTGTAAATTTTCATTGC	This study
Spr0693$_{T197M}$-F	GGATGCAATGACTGTTCTCAGTACCCT	This study
Spr0693$_{T197M}$-R	GAACAGTCATTGCATCCAATTGACTTTG	This study
Spr0693$_{I317M}$-F	CAACATGGAGGTTAAAAGCAAAACT	This study
Spr0693$_{I317M}$-R	TAACCTCCATGTTGACAGAAAAACCC	This study

Supplementary References

1. Hoskins, J. *et al*. Genome of the bacterium *Streptococcus pneumoniae* strain R6. *J. Bacteriol.* **183**, 5709-5717 (2001).
2. Lu, L., Ma, Y.Y. & Zhang, J.R. *Streptococcus pneumoniae* recruits complement factor H through the amino terminus yof CbpA. *J. Biol. Chem.* **281**, 15464-15474 (2006).
